# Function, Architecture, and Biogenesis of Reovirus Replication Neoorganelles

**DOI:** 10.3390/v11030288

**Published:** 2019-03-21

**Authors:** Raquel Tenorio, Isabel Fernández de Castro, Jonathan J. Knowlton, Paula F. Zamora, Danica M. Sutherland, Cristina Risco, Terence S. Dermody

**Affiliations:** 1 Cell Structure Laboratory, National Center for Biotechnology, CNB-CSIC, Cantoblanco Campus, 28049 Madrid, Spain; rtenorio@cnb.csic.es (R.T.); ifernandez@cnb.csic.es (I.F.d.C.); 2 Department of Pathology, Microbiology, and Immunology, Vanderbilt University School of Medicine, Nashville, TN 37232, USA; jonathan.knowlton@Vanderbilt.Edu; 3 Department of Microbiology and Molecular Genetics, University of Pittsburgh School of Medicine, Pittsburgh, PA 15219, USA; pfz3@pitt.edu; 4 Department of Pediatrics, University of Pittsburgh School of Medicine, Pittsburgh, PA 15224, USA; danica.sutherland@chp.edu; 5 Center for Microbial Pathogenesis, UPMC Children’s Hospital of Pittsburgh, Pittsburgh, PA 15224, USA

**Keywords:** reovirus, rotavirus, bluetongue virus, double-stranded RNA, viral factories, viral inclusions, viral replication organelles, endoplasmic reticulum, viral nonstructural proteins

## Abstract

Most viruses that replicate in the cytoplasm of host cells form neoorganelles that serve as sites of viral genome replication and particle assembly. These highly specialized structures concentrate viral proteins and nucleic acids, prevent the activation of cell-intrinsic defenses, and coordinate the release of progeny particles. Reoviruses are common pathogens of mammals that have been linked to celiac disease and show promise for oncolytic applications. These viruses form nonenveloped, double-shelled virions that contain ten segments of double-stranded RNA. Replication organelles in reovirus-infected cells are nucleated by viral nonstructural proteins µNS and σNS. Both proteins partition the endoplasmic reticulum to form the matrix of these structures. The resultant membranous webs likely serve to anchor viral RNA–protein complexes for the replication of the reovirus genome and the assembly of progeny virions. Ongoing studies of reovirus replication organelles will advance our knowledge about the strategies used by viruses to commandeer host biosynthetic pathways and may expose new targets for therapeutic intervention against diverse families of pathogenic viruses.

## 1. Introduction

Viruses are obligate intracellular pathogens that require host cells in order to replicate and produce infectious progeny. Virus entry into host cells is followed by capsid uncoating, genome transcription and replication, synthesis of viral proteins, assembly of progeny virions, and egress. For most viruses, genome replication and assembly take place in specialized intracellular compartments known as viral factories or inclusions [1,2,3,4], which are often composed of membranous scaffolds, viral and cellular factors, and mitochondria [5]. Viral inclusions (VIs) serve multiple purposes during infection, including the concentration of viral and host factors to ensure the high efficiency of replication, sequestration of viral nucleic acids and proteins from innate immune responses, and the spatial coordination of consecutive replication cycle steps [1,4,5]. Most double-stranded RNA (dsRNA) viruses form cytoplasmic inclusions with a characteristic morphology. These neoorganelles constitute sites of genome replication and virion assembly, and contain abundant viral RNA and proteins [6,7,8].

The combination of ultrastructural and functional studies has enhanced our knowledge about VI biogenesis. However, for many viruses, it is still not known how these structures form and mediate functions in viral replication. Here, we describe the current understanding of the morphogenesis and function of reovirus inclusions and compare these neoorganelles with the replication factories formed by other members of the *Reoviridae* family.

## 2. Composition of Reovirus Inclusions

**Figure 1:**
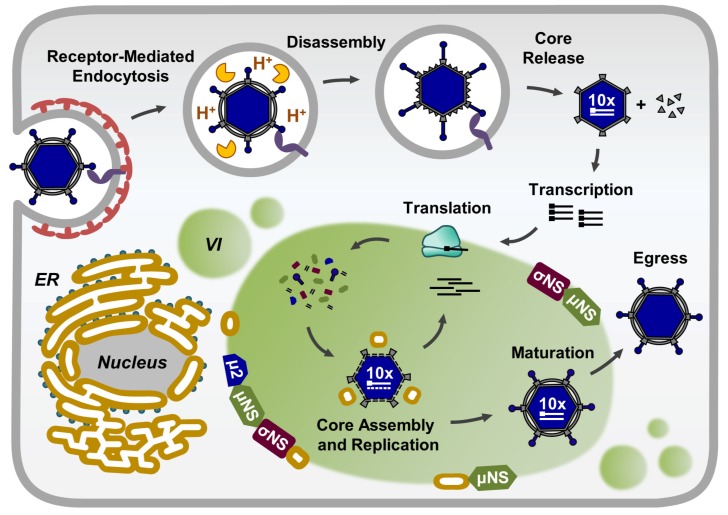
The reovirus replication cycle. VI—viral inclusions; ER—endoplasmic reticulum.

Mammalian orthoreoviruses (called reoviruses here) are nonenveloped, icosahedral viruses consisting of two concentric protein shells—an outer capsid and inner core—that contain a genome of ten dsRNA segments [9]. The reovirus replication cycle is entirely cytoplasmic (Figure 1). Following entry into the cells, the viral outer capsid is proteolytically cleaved in the late endosomes to form transcriptionally active cores [10,11,12]. These particles are released into the cytoplasm and synthesize full-length, positive-sense, capped, and nonpolyadenylated single-stranded RNAs (ssRNAs) corresponding to each viral genome segment [13,14]. Reovirus ssRNAs are translated to synthesize new viral proteins and also serve as templates for negative-sense ssRNA synthesis within the replicase particles to produce nascent genomic dsRNA [15,16]. The addition of outer-capsid proteins onto newly formed viral cores completes the reovirus assembly process.

In a variety of cell types, reovirus inclusions can be detected by light microscopy as early as 4 h post-infection. VIs contain several types of filaments, viral proteins, ssRNAs, dsRNAs, and viral particles at various stages of morphogenesis [17,18,19,20,21]. At late times post-infection, VIs are filled with mature virions arranged in paracrystalline arrays [19,22,23]. Reovirus nonstructural proteins μNS and σNS and structural protein μ2 function in the formation and structural organization of reovirus inclusions [24,25]. Specific interactions between μ2 and μNS are required to form VIs and to recruit the additional viral (and perhaps host) factors that mediate viral genome replication and assembly [26,27]. High-resolution structures of the inclusion-forming proteins (μNS, σNS, and μ2) are not available. This information would help us to better understand the interactions between these (and other viral and host) proteins during inclusion biogenesis. Ultrastructural studies of reovirus inclusions show that these structures contain macromolecular complexes, ribosomes, and microtubules [23,28,29], indicating that these neoorganelles are rich in the cellular components required for viral progeny production.

**Figure 2:**
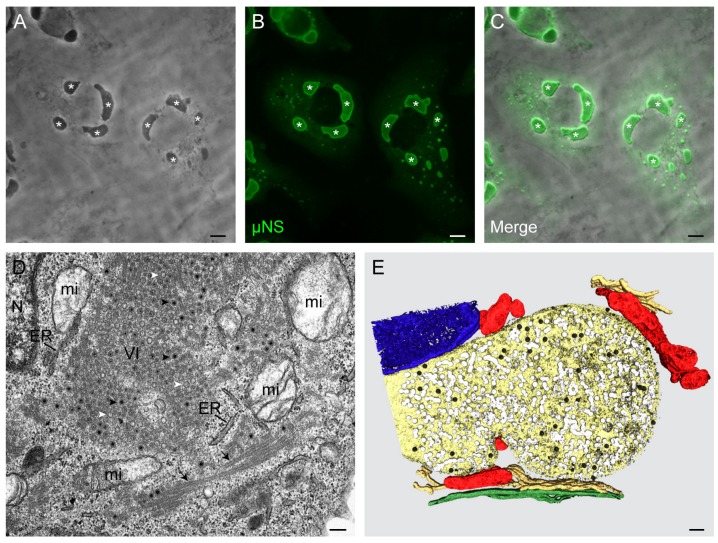
Reovirus inclusions imaged by light and electron microscopy. (**A**–**C**) Human brain microvascular endothelial cells were infected with reovirus strain T1L M1-P208S for 24 h, and were fixed, permeabilized, and processed for immunofluorescence staining with a chicken anti-μNS polyclonal serum and a secondary antibody conjugated with Alexa 594 (green). This strain forms large, globular VIs. (**A**) The phase-contrast microscopy shows dark, dense, globular structures (asterisks) in the cytosol of reovirus-infected cells. (**B**) The localization of μNS by fluorescence microscopy confirms that the dense structures seen by phase-contrast microscopy are viral inclusions (asterisks). (**C**) The merging of phase-contrast and fluorescence microscopy images. (**D**,**E**) HeLa cells were infected with T1L M1-P208S and fixed at 24 h. (**D**) Ultrathin sections (~70 nm) of infected cells were imaged by transmission electron microscopy (TEM). A characteristic viral inclusion (VI) is shown. The VI contains mature virions (black arrowheads) and empty viral particles (white arrowheads). Mitochondria (mi), endoplasmic reticulum (ER) cisternae, and microtubules (arrows) surround the VI. N—nucleus. (**E**) VI as visualized by TEM of serial sections, 3D reconstruction, and image processing. The mitochondria (red) and ER cisternae (gold) surround a network of smooth membranes (light yellow) with mature virions (black) and empty viral particles (white). The nucleus is colored in blue and the microtubules in green. Scale bars are 10 μM in (**A**–**C**) and 250 nm in (**D**,**E**).

Until recently, it was thought that reovirus inclusions did not contain membranes. However, studies employing sophisticated light and transmission electron microscopy (TEM) revealed that VIs are formed from membranes [30,31]. Transmission electron micrographs of oriented serial sections and three-dimensional (3D) reconstructions of the reovirus inclusions demonstrated that these neoorganelles contain membranous scaffolds and are surrounded by mitochondria, lipid droplets, and endoplasmic reticulum (ER) cisternae [30]. Viral proteins, dsRNAs, and viral particles appear to adhere to the membranes inside the inclusions [31]. The high concentration of macromolecular complexes, viral particles, and membranes of VIs might explain the high density of these structures compared with that of the cytosol, which is consistent with the highly refractive nature of these structures, as observed by phase-contrast microscopy (Figure 2).

## 3. Morphology and Functions of Reovirus Inclusions

Following entry into the cells, reovirus ssRNAs are released into the cytoplasm from transcribing core particles and are translated, yielding eleven reovirus proteins (eight structural and three nonstructural proteins) [32]. The viral nonstructural protein μNS, one of the first viral proteins synthesized, plays a crucial role in the biogenesis of VIs. The μNS protein expressed alone can form globular VI-like structures, which grow and fuse like the VIs in reovirus-infected cells [25,33,34]. These observations suggest that μNS is the viral protein responsible for inclusion nucleation [26]. In turn, the μNS protein recruits viral core proteins and the nonstructural protein σNS, which is a key factor for viral RNA replication [35,36,37]. The μ2 protein, which is a minor constituent of the viral core, defines the morphology of VIs. Most reovirus strains produce filamentous inclusions, which are attributable to the interactions of μ2 with microtubules and the stabilization of the microtubule network [27]. Inside cells, the amino-terminal domain of μ2 associates with microtubules, and the carboxy-terminal domain binds to μNS [38]. However, some laboratory isolates of reovirus strain type 3 Dearing, which is commonly used for studies of reovirus replication and pathogenesis [9], produce globular inclusions [39]. The globular shape of these VIs is due to a single mutation in μ2, P208S, which abrogates its capacity to interact with microtubules [27]. The μNS protein also associates with microtubules and mediates inclusion movement and enlargement during infection. Both filamentous and globular inclusions rely on an intact microtubule network for VI assembly, maintenance, and dynamics [34]. Thus, small inclusions track along microtubules and coalesce to form large perinuclear structures [25,27,34,40].

The cytoskeleton also participates in reovirus genome packaging. VIs formed by reovirus strains deficient in microtubule-binding accumulate relatively more empty (genome-lacking) viral particles, while microtubule-binding reovirus strains form VIs that have a higher percentage of complete (genome-containing) virions [40]. Interestingly, the inefficient genome packaging observed with the strains deficient in microtubule-binding can be ameliorated by rerouting the viral factories to the actin cytoskeleton [40], suggesting that viral inclusions can track along different cytoskeletal filaments. Reovirus also usurps vimentin intermediate filaments, which are reorganized during infection [20]. Thus, reovirus uses the cellular cytoskeleton to facilitate VI functions.

Within inclusions, the reovirus core particles synthesize viral RNAs. The reovirus RNA-dependent RNA polymerase (RdRp) is composed of one subunit of λ3, which is responsible for the catalytic activity, and two subunits of μ2, which function as cofactors [29,41]. The reovirus polymerase catalyzes fully conservative transcription using negative-strand RNAs as templates for positive-strand RNA synthesis [42]. Nascent positive-strand RNAs are capped and methylated by the viral λ2 protein during release from the core [43]. The parental positive-sense RNA strand re-anneals with the negative-sense RNA strand to reform the dsRNA genome. Thus, VIs protect viral mRNAs from the cytoplasmic environment to favor viral transcription.

The nonstructural protein σNS, an RNA-binding protein with a strong affinity for ssRNAs, is essential for inclusion development and viral replication. During infection, σNS is found within the VIs and co-localizes with μNS at the periphery of these structures [44]. In contrast to μNS, σNS appears to diffuse in the cytoplasm when expressed alone and localizes to inclusions through its interactions with μNS [36]. The first eleven amino acids of σNS, which contain several positively charged residues, are required for σNS distribution to the inclusions [36]. The σNS protein binds and stabilizes the viral RNAs, which might be necessary for sequestering the viral transcripts inside the inclusions. This function of σNS might also protect these RNAs from cytoplasmic nucleases, prevent the activation of innate immune responses, and facilitate viral translation [37]. Additionally, σNS likely recruits the translational machinery to VIs, as σNS co-localizes with eukaryotic translation initiation factor 3 subunit A, ribosomal P protein, phosphorylated ribosomal protein S6, and ribosomal protein S3 in the reovirus inclusions [28]. Inside these structures, σNS also co-localizes with ER proteins, suggesting that σNS couples the host translational machinery to the sites of particle morphogenesis [28]. Moreover, σNS functions in stress granule disassembly, which could amplify its role as a translational activator [45].

Viral translation also is enhanced by the reovirus σ3 outer-capsid protein [46]. The σ3 protein binds dsRNA during infection, blocking the activation of protein kinase RNA-activated (PKR), an interferon-induced enzyme that is activated by binding to dsRNA [47]. Activated PKR phosphorylates and thus inhibits the eukaryotic translation initiation factor 2 subunit α, an essential translation initiation factor, resulting in the suppression of host protein synthesis [48,49,50]. These effects may contribute to the preferential synthesis of reovirus proteins in or near the inclusions where σ3 is abundant [51]. Reovirus infection also impairs the function of interferon regulatory factor 3, a transcription factor required for the induction of an antiviral state, by sequestering it inside the inclusions via interactions with μNS [52]. Thus, several viral proteins interact intimately with the host machinery to promote replication steps within VIs, and impede innate immune activation.

## 4. Hsp70, Hsp90, and the TRiC Chaperonin are Required for Reovirus Assembly

In addition to remodeling host membranes, engaging the cytoskeleton, and commandeering the translational machinery to form functional VIs, reovirus recruits an array of protein-folding chaperones that participate in viral assembly. Heat shock protein 70 (Hsp70), heat shock protein 90 (Hsp90), and the T-complex protein-1 (TCP-1) ring complex (TRiC; also called CCT for chaperonin containing TCP-1) chaperonin fold several reovirus proteins.

Hsp70 and Hsp90 are protein-folding chaperones that bind and stabilize nascent polypeptide chains during or immediately after translation, in an ATP-dependent manner [53,54]. Both chaperones facilitate the assembly of the reovirus σ1 outer-capsid protein, an event that likely occurs inside the VIs as the last step in reovirus particle assembly. The σ1 protein is a homotrimer responsible for engaging cell-surface receptors [55]. The biogenesis of the σ1 trimer is a multistep process that involves a co-translational trimerization event at the amino-terminus and a post-translational Hsp70/90-dependent trimerization event at the carboxy-terminus [56]. A complex of Hsp70, Hsp90, and σ1 monomers may be the functional structures responsible for σ1 trimerization [57]. If present, this complex may exist at spatially defined locations within the VIs to coordinate the final step in the assembly process. Along with Hsp70, another chaperone, the heat shock cognate protein 70 (Hsc70), is recruited to the VIs. Hsc70 is anchored to the VIs by interactions with the μNS protein [58], highlighting a potential function for μNS in concentrating the folding machinery required for assembly.

The TRiC chaperonin serves an essential function in reovirus replication by folding the major outer-capsid protein σ3. TRiC is a large, one-megadalton protein complex composed of two identical eight-member rings stacked back-to-back, forming a central cavity that catalyzes protein folding [59,60]. TRiC is ubiquitous in eukaryotes and functions as a highly specialized chaperone that folds essential substrates, including actin and tubulin [61,62]. The σ3 protein is a structural component of the viral outer capsid that complexes with the reovirus μ1 protein, forming a heterohexamer composed of three σ3 molecules and three μ1 molecules. TRiC redistributes to the VIs in reovirus-infected cells and folds σ3 into its native conformation [63]. The exact mechanism by which TRiC folds σ3 is not clear, and it is not known whether TRiC participates in the assembly of the σ3–μ1 heterohexamer. Interestingly, TRiC also is required for proper VI morphogenesis [63]. The viral outer-capsid proteins are likely translated in close proximity to the inclusions and may assemble onto nascent core particles in a mechanism involving TRiC or another chaperone complex. Therefore, multiple chaperone networks cooperate to assemble nascent reovirus particles within VIs.

## 5. Viral Inclusions are Formed from Remodeled ER Membranes

**Figure 3:**
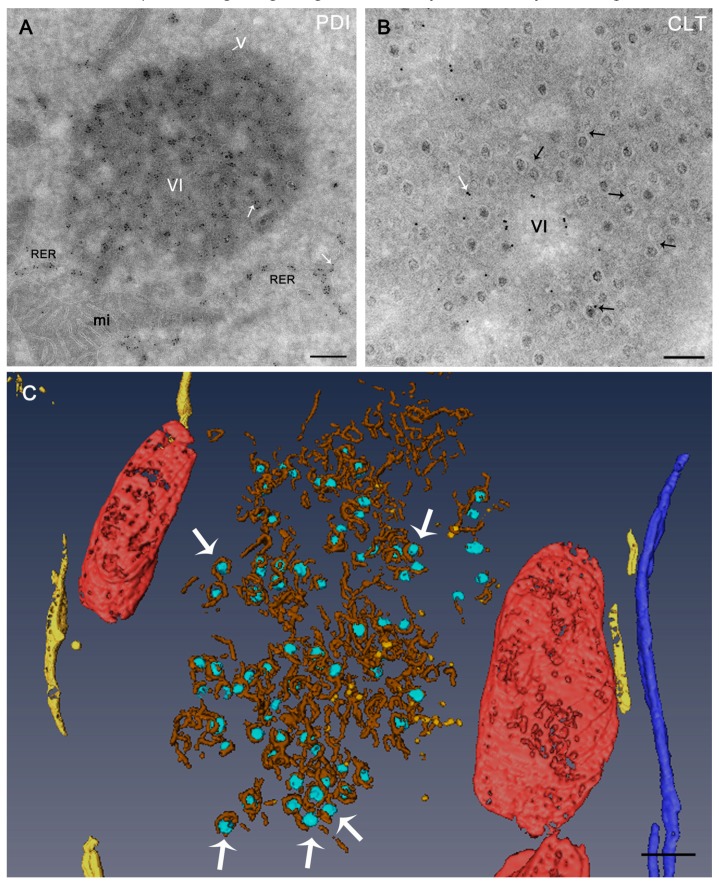
Reovirus inclusions contain ER membranes. (**A**,**B**) HeLa cells were infected with reovirus T1L M1-P208S for 14 h, frozen in liquid nitrogen, and sectioned at −120 °C. The thawed cryosections were processed for immunogold labeling with primary antibodies specific for two ER proteins—protein disulfide isomerase (PDI) (**A**) and calreticulin (CLT) (**B**)—and for secondary antibodies conjugated with 10 nm colloidal gold particles. The rough ER (RER) cisternae around the VIs and membranes inside the VIs are labeled with antibodies specific for ER proteins (white arrows in **A** and **B**). These membranes are in close contact with the viral particles (black arrows in **B**). V—viral particle. (**C**) Electron tomography (ET) of a single VI. A thawed cryosection was processed by single-tilt-axis ET, 3D reconstruction, and image processing. The 3D model shows that the VI is a collection of vesicles and tubules with viral particles attached to membranes (white arrows). RER—yellow; viral particles—light blue; mitochondria—red; nuclear membrane—dark blue; tubules and membrane fragments inside the inclusion—brown; vesicles inside the inclusion—orange. Scale bars are 500 nm in (**A**) and 200 nm in (**B**,**C**). Modified from Tenorio et al., 2018 [31].

State-of-the-art imaging methods have led to the discovery that membranes within the reovirus VIs are derived from the ER. Using light and electron microscopy, ER-specific markers are observed in VI membranes [31]. The immunogold labeling of thawed cryosections, a technique known as the Tokuyasu method, shows the specific labeling of ER proteins in VI membranes (Figure 3A,B). Because of the lack of a dehydration step, this method provides the optimal preservation of membranes and epitopes [64,65]. Electron tomography (ET) of Tokuyasu cryosections showed the fine details of the internal organization of the VIs. Interestingly, the viral particles inside the VIs are attached to the membranes (Figure 3C). This inner membranous network within the VIs may provide physical support for processes such as translation, genome replication, and progeny particle assembly [9,28,43]. Based on studies localizing viral RNA synthesis with BrU, it appears that reovirus genome replication and secondary rounds of transcription occur within newly synthesized cores associated with ER-derived membranes [31]. There is no evidence that reovirus buds into the lumen of a membrane-bound compartment to obtain its outer-capsid proteins. However, given that the µ1 outer-capsid protein is myristoylated [66], it is also possible that inclusion-associated membranes function in µ1 folding, targeting, or assembly onto newly forming virions.

**Figure 4:**
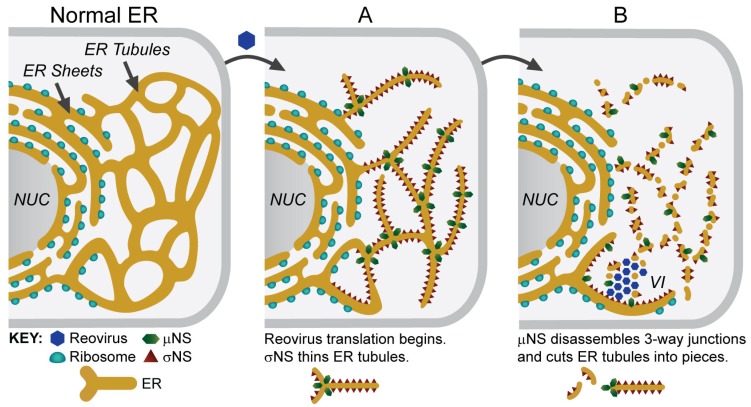
Model of ER remodeling and VI biogenesis. The ER in uninfected cells is composed of sheets and tubules. (**A**) In reovirus-infected cells, σNS binds to the ER tubules and transforms them into thin structures. (**B**) µNS binds to these thin tubules and triggers their fragmentation. Small tubules and vesicles coalesce to form the VI. The schematics at the bottom demonstrate how σNS and µNS might remodel the ER. NUC—nucleus. Modified from Tenorio et al., 2018 [31].

The membranous matrix of reovirus VIs derives from the extensive remodeling of the peripheral ER [31]. Peripheral ER elements become thinner, fragment, and partially aggregate throughout the course of reovirus infection. These changes are observed at very early stages of infection, in both fixed and living cells [31]. Confocal microscopy and stimulated emission depletion (STED) super-resolution microscopy revealed that viral nonstructural proteins σNS and µNS localize in close proximity to the ER in reovirus-infected cells. The remarkable changes in the ER architecture that occur during infection can be reproduced by ectopically expressing σNS and µNS. σNS causes ER tubulation, whereas µNS causes ER vesiculation [31] (Figure 4). It is not known whether this extensive remodeling triggers an ER stress responses or affects ER function. Future studies should clarify these points.

Metal-tagging TEM (METTEM) is a sensitive method for molecular mapping in situ [67,68]. This technique uses the metal-binding protein metallothionein (MT) as a tag for TEM. MT binds gold atoms in vivo and builds an electron-dense nanoparticle that is easily visible by TEM. METTEM of reovirus-infected cells revealed that µNS attaches to the remodeled ER tubules and vesicles before VIs are formed [31]. These observations suggest that µNS mediates ER vesiculation by direct interaction with ER membrane components.

## 6. Viruses and the ER

The ER is rearranged by many viruses to facilitate different steps in viral replication [69]. Members of the *Bromoviridae*, *Flaviviridae*, and *Tombusviridae* families transform the ER into vesicles, invaginations, or spherules [70,71,72,73]. Viruses in other families, such as the *Arteriviridae*, *Coronaviridae*, *Flaviviridae*, and *Picornaviridae,* use ER membranes to build single-membrane tubules or double-membrane vesicles [74,75,76,77]. Coronaviruses and flaviviruses also produce convoluted membranes from the ER [78,79], and gamma coronaviruses use the ER to form zippered membranes [80]. For reoviruses, a collection of ER tubules and vesicles associated with a network of modified ER cisternae form VIs. However, the mechanisms responsible for the ER remodeling observed in reovirus-infected cells are not clear.

As reovirus nonstructural proteins σNS and µNS lack predicted transmembrane domains, their effects on ER remodeling could be mediated by interfering with ER-shaping proteins such as atlastins, Rab GTPases, reticulons, or Lunapark [81,82,83]. These proteins are involved in the ER remodeling induced by other viruses, such as brome mosaic virus [84], hepatitis C virus [85], and enterovirus [86]. Lipid-transfer proteins also could be targeted by reovirus nonstructural proteins. The ER-resident vesicle-associated membrane protein (VAMP)-associated proteins and oxysterol-binding protein 1 exchange lipids between the ER and other organelles. These proteins modify the membrane composition and structure by regulating the ER–organelle contact sites, and they are usurped by some RNA viruses to stabilize the replication complexes [87,88,89].

Another possible mechanism for ER remodeling involves direct interactions between reovirus proteins with ER lipids. Several viral proteins interact with lipids in cell membranes and disrupt or modify their structures to build replication organelles [90]. However, there are few examples of proteins from nonenveloped viruses that directly interact with membrane lipids. Reovirus fusion-associated small transmembrane (FAST) proteins are expressed by avian and reptilian reoviruses as well as some reovirus isolates from bats. They are not expressed by mammalian orthoreoviruses. The FAST proteins bind to lipid rafts in the plasma membrane and function to approximate the adjacent cell membranes to induce cell-to-cell fusion [91]. These proteins are not thought to function in VI formation. How reovirus σNS and µNS proteins transform the ER to build VIs remains to be elucidated.

## 7. Comparison of Reovirus Inclusions to Other Members of the *Reoviridae*

Reoviruses represent one genus of the *Reoviridae* family and share a number of characteristics with other viruses in this family. Rotaviruses, which comprise another *Reoviridae* genus, also form large cytoplasmic inclusions, termed viroplasms, that house key viral replication steps. Like reovirus VIs, rotavirus viroplasms are dynamic structures that move to the perinuclear region during infection and fuse with each other [92]. The co-expression of rotavirus NSP5 with either NSP2 [93] or VP2 [92,94,95] in uninfected cells leads to the formation of viroplasm-like structures. Rotavirus NSP5 and NSP2 appear to have functions in inclusion biogenesis analogous to those of the reovirus σNS and μNS proteins. In addition, viroplasm assembly requires the phosphorylation of NSP5 and NSP2 by cellular casein kinase 1α (CK1α) [96,97,98,99]. The phosphorylation of NSP2 is essential for the protein to traffic to sites of viroplasm formation, which is most likely at cellular lipid droplets [100,101]. In rotavirus-infected cells, NSP5 is hyperphosphorylated by a CK1/2-dependent mechanism [96,98,99,102]. Interestingly, the form of NSP2 located within the viroplasm interacts only with hyperphosphorylated NSP5, and this interaction is required for viroplasm formation [98].

Much like reovirus VIs, microtubules are important components of rotavirus viroplasms and form complexes with NSP2 and structural proteins VP1 and VP2 [92,98,103]. Viroplasm morphogenesis depends on other components of the host machinery, such as the proteasomes and elements of the autophagy pathway [104,105,106]. Lipid droplets also might have a function in viroplasm formation, as lipid droplet-associated proteins co-localize with rotavirus viroplasms during infection [100,107]. Interestingly, reovirus VIs are frequently surrounded by lipid droplets [30], but their role in reovirus infection is not known. Some nuclear factors also redistribute to viroplasms during infection. Nuclear hnRNPs and AU-rich element-binding proteins, nuclear transport proteins, and some cytoplasmic proteins directly interact with the viroplasmic NSP2 and NSP5 proteins in an RNA-independent manner and become sequestered in the viroplasms of infected cells [108].

In contrast to reovirus VIs, rotavirus viroplasms are not thought to contain ER membranes, although ultrastructural imaging studies like those conducted for reovirus have not been reported. Nonetheless, rotavirus uses ER membranes during particle maturation and assembly. The incorporation of viral outer-capsid proteins onto nascent virions occurs in the ER lumen and not in viroplasms and is mediated by rotavirus NSP4, a transmembrane glycoprotein that mainly distributes to ER membranes [109,110]. Following the assembly of the outer capsid, the fully formed virions exit the ER and are transported to the cell surface using small smooth vesicles [111]. These observations suggest that the use of ER membranes at different steps of infection is a common feature of the *Reoviridae*.

Bluetongue virus (BTV) is the prototype member of the *Orbivirus* genus of the *Reoviridae* family. The BTV NS2 protein is the principal component of viral inclusion bodies, which are equivalent to the VIs of reovirus and viroplasms of rotavirus [112,113]. NS2 recruits the viral ssRNAs and protein components required for core assembly and genome replication [114,115,116]. NS2 may be a μNS homolog [114,117]. Similar to rotavirus, the BTV outer-capsid proteins are not recruited to the viral inclusion bodies. Instead, they interact with host factors such as the soluble N-ethylmaleimide-sensitive-factor attachment protein receptor (SNARE) regulatory protein synaptotagmin I [118], vimentin [119], and endosomal sorting complex required for trafficking (ESCRT) [120] in the cytoplasm. This process appears to be coupled to a non-lytic, exocytic pathway [121]. As in reovirus and rotavirus infection, host proteins modulate the dynamics and function of the BTV inclusion bodies. Casein kinase 2 and protein phosphatase 2A are host enzymes that regulate inclusion morphology and BTV replication [122]. Contrary to reovirus and rotavirus, the microtubule network does not appear to be involved in the morphogenesis of the inclusion bodies formed by BTV [115]. Therefore, microtubules are not essential for the replication of all members of the *Reoviridae* family.

Avian reoviruses belong to the genus *Orthoreovirus* and also replicate in cytoplasmic inclusions with a globular morphology [123,124]. Avian reovirus inclusions are not microtubule-associated and are formed by nonstructural protein μNS [125]. Analogous to mammalian orthoreovirus μNS, avian reovirus μNS is the minimal viral factor required for inclusion formation during avian reovirus infection [126]. Avian reovirus σNS is homologous to mammalian orthoreovirus σNS, and both proteins bind RNA [127]. Avian reovirus assembly occurs exclusively within cytoplasmic inclusions, starting with the selective recruitment of σNS and structural protein λA to small μNS-containing inclusions [126].

Collectively, these studies suggest that the inclusions formed by members of the *Reoviridae* family share some characteristics related to their composition and structure. Viral proteins and frequently cytoskeletal elements have an essential role in the first steps of inclusion formation. In addition, different steps of the replication cycle of *Reoviridae* viruses are associated with membranes and cellular organelles, such as the ER, which functions in reovirus and rotavirus replication. Structural and biochemical studies are required in order to determine whether the viral inclusions formed by other members of the *Reoviridae* contain membranous scaffolds, as is the case for reovirus.

## 8. Conclusions

Relative to the initial steps of reovirus infection (receptor engagement and cell entry), less is known about the later replication steps, especially those required for the formation of viral replication organelles (Table 1). These neoorganelles are specialized structures required for productive viral infection and represent the morphological rewiring of host cells to foster the assembly of thousands of progeny viral particles. A common strategy used to build these structures by viruses with cytoplasmic replication programs involves the establishment of membranous scaffolds, but the precise scaffolding mechanisms vary by virus and are not entirely understood. Mammalian orthoreovirus employs a strategy of ER fragmentation to build replication organelles that are dependent on viral nonstructural proteins σNS and µNS and structural protein µ2. As facets of this replication strategy may be conserved across virus families, understanding which host factors are required for reovirus inclusion formation (e.g., ER-shaping proteins), capsid assembly (e.g., protein-folding networks), and egress of viral progeny, may be broadly applicable. The identification of these key viral and cellular factors involved in the biogenesis and function of viral replication organelles will enhance the knowledge of basic cell biology and may illuminate new targets for antiviral drug development.

## Figures and Tables

**Table 1:** Key unanswered questions in studies of reovirus replication organelles. ER—endoplasmic reticulum; VI—viral inclusion.

(1) How do the reovirus σNS and μNS proteins remodel the ER?
(2) How does ER remodeling affect its function?
(3) What is the role of VI membranes in reovirus replication and morphogenesis?
(4) How do reovirus proteins interact with host factors to promote viral replication and morphogenesis?
(5) What are the precise functions of σNS and μNS inside VIs?
(6) Do other members of the *Reoviridae* assemble membranous replication neoorganelles?
(7) How do mitochondria become recruited to and interact with VIs?
(8) How do chaperone networks participate in the morphogenesis of reovirus particles?
(9) What mechanisms are used by reovirus to exit infected cells?
(10) Can the host factors required for reovirus inclusion formation and morphogenesis be targeted by small molecules as an antiviral strategy?
